# Supplementary figures and images for: Identification of iso-diabolic acid-based tetraester and mixed ether/ester membrane-spanning lipids in members of the Bacillota provides insight into the biosynthesis of bacterial branched glycerol dialkyl glycerol tetraethers
Source: Appl Environ Microbiol. 2026 May 18;92(6):e00289-26. doi: 10.1128/aem.00289-26 (PMC13274423; doi:10.1128/aem.00289-26)

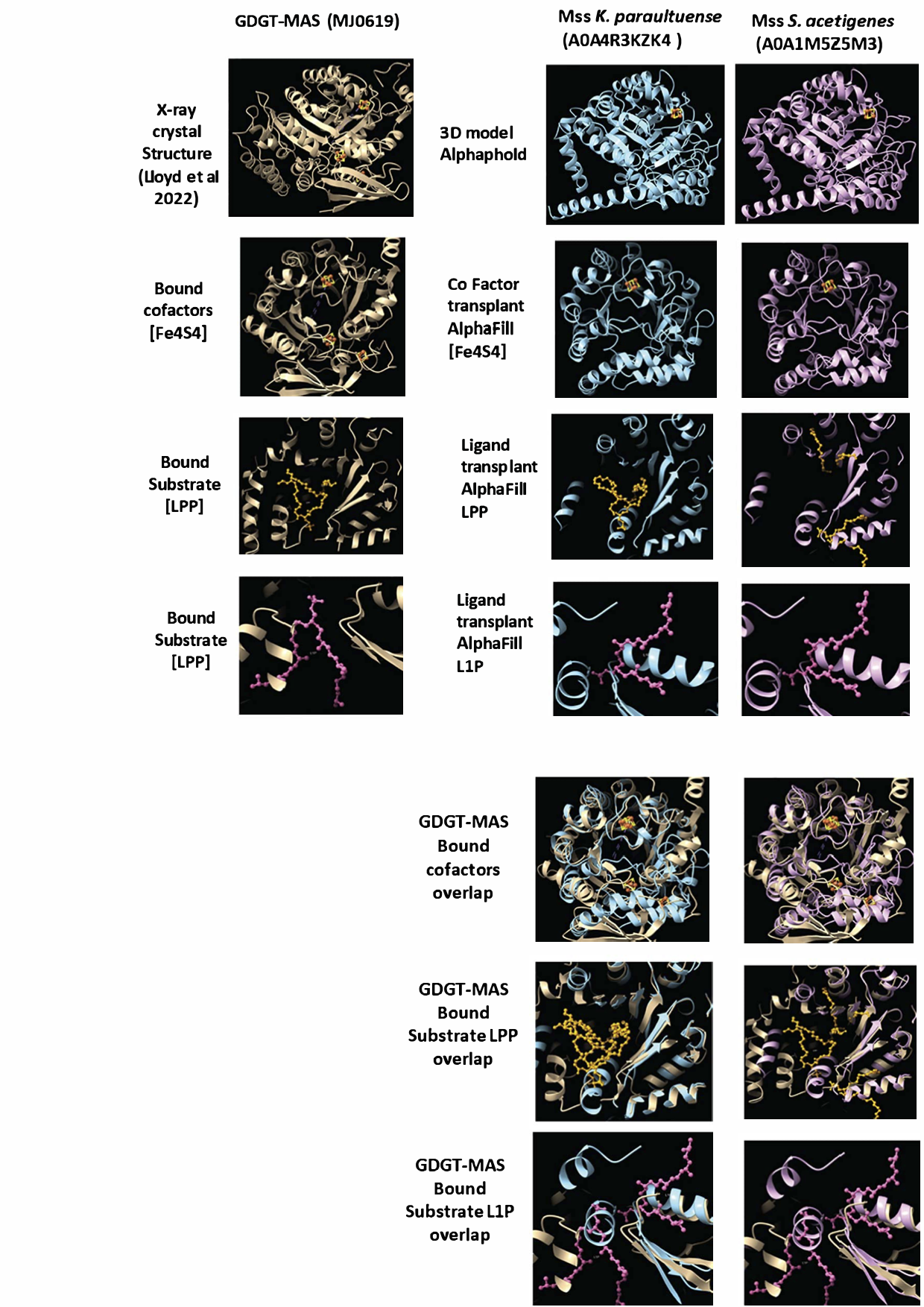

Supplement: Figure S1 — Architecture and tridimensional structure of the membrane-spanning lipid enzymes from the archaeal Tes synthase and bacterial Mss homologs. [file aem.00289-26-s0001.tiff]
