# Supplementary material for: Identification of iso-diabolic acid-based tetraester and mixed ether/ester membrane-spanning lipids in members of the Bacillota provides insight into the biosynthesis of bacterial branched glycerol dialkyl glycerol tetraethers
Source: Appl Environ Microbiol. 2026 May 18;92(6):e00289-26. doi: 10.1128/aem.00289-26 (PMC13274423; doi:10.1128/aem.00289-26)
Supplement: Supplemental legend — Descriptive legend for Fig. S1. [file aem.00289-26-s0002.docx]

**Supplementary Figure 1.** Architecture and tridimensional structure of the Membrane-spanning lipid enzymes from the archaeal Tes synthase (also known as GDGT-MAS; MJ0619) and bacterial Mss homologs. Comparison of the MJ0619 reference with bacterial Mss homologs. Left column: X-ray structure of Tes synthase/GDGT-MAS (59) shows the radical-SAM core containing three [4Fe–4S] (SF4) cofactors bound alongside the lipid substrates phosphatidic acid (LPP) and the archaeal lipid 2,3-di-*O*-phytanyl-sn-glycero-1-phosphate (L1P) . Middle column: AlphaFold model of Mss from *K. paraultunense* (UniProt A0A4R3KZK4) incorporating AlphaFill SF4 cofactors and transplanted LPP/L1P substrates. Right column: AlphaFold model of Mss from *S. acetigenes* (UniProt A0A1M5Z5M3) with AlphaFill SF4 cofactors and LPP/L1P substrates. Proteins are displayed as cartoons; cofactors and ligands are shown as stick models. Bottom rows: Structural superposition onto GDGT-MAS (ChimeraX MatchMaker) demonstrates that both Mss enzymes position their radical-SAM [4Fe–4S] cluster and lipid substrates within similar hydrophobic tunnels, with the polar head groups extending toward the solvent interface. The conserved arrangement of cofactors and lipid tails indicates a common catalytic zone and substrate binding site consistent with the radical-SAM mediating tail-to-tail condensation of membrane lipid chains.
